# Studied of Defatted Flour and Protein Concentrate of *Prunus serotine* and Applications

**DOI:** 10.3390/foods9010029

**Published:** 2019-12-27

**Authors:** Analía A. Lu Martínez, Juan G. Báez González, Minerva Bautista Villarreal, Karla G. García Alanis, Sergio A. Galindo Rodríguez, Eristeo García Márquez

**Affiliations:** 1 Universidad Autónoma de Nuevo León, Facultad de Ciencias Biológicas, Departamento de Alimentos, Avenida Universidad s/n, Ciudad Universitaria, San Nicolás de los Garza, NL 66455, Mexico; liamtz18@hotmail.com (A.A.L.M.); minevillareal@hotmail.com (M.B.V.); karla_alanis23@hotmail.com (K.G.G.A.); 2 Universidad Autónoma de Nuevo León, Facultad de Ciencias Biológicas, Laboratorio de Nanotecnología, Avenida Universidad s/n, Ciudad Universitaria, San Nicolás de los Garza, NL 66455, Mexico; sagrod@yahoo.com.mx; 3 Centro de Investigación y Asistencia en Tecnología y Diseño del Estado de Jalisco, Autopista Mty-Aeropuerto Km 10 Parque PIIT, Vía de Innovación 404, Apodaca, NL 66629, Mexico

**Keywords:** *Prunus serotine*, defatted flour, soluble protein, protein concentrate, emulsifying properties, emulsion stability

## Abstract

*Prunus serotine* seed, was processed to produce a defatted flour (71.07 ± 2.10% yield) without hydrocyanic acid. The total protein was 50.94 ± 0.64%. According to sensory evaluation of cookies with *P. serotine* flour, the highest score in overall impression (6.31) was at 50% flour substitution. Its nutritional composition stood out for its protein and fiber contents 12.50% and 0.93%, respectively. Protein concentrate (*Ps*PC) was elaborated (81.44 ± 7.74% protein) from defatted flour. Emulsifying properties of *Ps*PC were studied in emulsions at different mass fractions; ϕ = 0.002, 0.02, 0.1, 0.2, and 0.4 through physicochemical analysis and compared with whey protein concentrate (WPC). Particle size in emulsions increased, as did oil content, and results were reflected in microscope photographs. *Ps*PC at ϕ 0.02 showed positive results along the study, reflected in the microphotograph and emulsifying stability index (ESI) test (117.50 min). At ϕ 0.4, the lowest ESI (29.34 min), but the maximum emulsifying activity index (EAI) value (0.029 m^2^/g) was reached. WPC had an EAI value higher than *Ps*PC at ϕ ≥ 0.2, but its ESI were always lower in all mass fraction values. *Ps*PC can compete with emulsifiers as WPC and help stabilize emulsions.

## 1. Introduction

Nowadays, there is an increasing demand for products of high nutritional quality [1]. Proteins are one of the major components of the human diet because of their nutritional properties. They are also responsible for physicochemical properties such as solubility, water, and oil retention capacity, foaming and emulsifying capacity, viscosity, and gelation, among others. The proteins impact not only the quality of the products, but also acceptance by consumers [2].

Protein is available in a variety of dietary sources [3]. In recent years, the growing concern of consumers with respect to animal safety has forced the industry to use vegetable proteins [4,5,6]. This type of proteins has health benefits, e.g., reduction of blood cholesterol levels, prevention of obesity and lower risk of heart diseases and cancer [7]. Vegetable proteins, when mixed with cereals, provide an alternative source of amino acids [3], which is why enrichment of other protein sources such as oilseeds and legumes with cereal-based foods has received considerable attention [8].

Baked snacks, such as bread and cookies, are widely accepted and consumed throughout the world and have become an attractive target for feeding and nutritional status improvement programs. This is especially true for cookies, because they not only offer a good vehicle for protein enrichment for consumers, but also because of their wide-spread consumption (5.9 per capita in 2019) and long shelf life [9,10,11].

The implementation of wheat flour substitutes or mimicry are desirable alternatives to achieve not only a decrease in calories, but also, to obtain a healthier nutritional profile in their composition [12,13]. Legumes and oilseeds such as soy, sunflower, barley, melon seeds, peanuts, hazelnuts, walnuts, sesame seeds, cashews, and almonds, are some alternative sources of flour [9,10].

Also, food grade films, hydrogels, foams, and emulsifiers have been developed from vegetable proteins. Emulsions are capable of absorbing at the oil-in water interface or air-in water dispersion [7,14]. These are part of many processed food formulations. Proteins are widely used for encapsulation of active substances. The proteins are used as a wall material around the active principle droplet, manifesting advantages such as biocompatibility, biodegradability, amphiphilic and hydrophobic and functional properties [15]. Moreover, vegetable proteins can be combined with other polymers, forming a variety of complexes with different structures (e.g., double networks, mosaic textures and cross-linked structures) [7].

In emulsions, the emulsifying activity index, emulsifying stability index, droplet size, interfacial properties and viscosity parameters are used [1]. Other techniques that help to understand the structure of the emulsions and morphology of the particles, particle size, and colloid instabilities (e.g., flocculation, aggregation) are light microscopy, SEM, and dynamic or static light scattering [16]. Among the vegetable proteins emulsifiers options, we found mainly leguminous foodstuffs like soy, lupin, peas, and chickpeas, cereals like wheat, barley, corn, and rice and oil seed such as peanuts, sunflowers, canola, flaxseed, and sesame. [7,17].

In Mexico, the oilseed *Prunus serotine* is widely distributed, and can be found in 16 states of the Republic. Nowadays the production of the fruit goes to 467.96 tons per year [18,19]. However, only the fruit and leaves have been used since colonial times for nourishment and medicinal purposes [20]. While the seed is still of little economic value because of the waste of its nutritional benefits, since it is only consumed as a toasted snack, the main nutrients in its composition are unsaturated fatty acids (89.9%) such as oleic, linoleic, and α-eleostearic acid, crude fiber (10.73 ± 1.49%) and protein (37.95 ± 0.16%) with 88.12 ± 0.72% of digestibility [18,21]. A protein value higher than other oilseeds like *P. dulcis* (19.91%) and *Arachis hypogaea* (22.82%), having lysine as the limiting amino acid. It has also been reported that digestibility values higher than 80% are related to an efficient amino acid bioavailability [18].

Its oil composition is also considered unique because of the significant content of α-eleostearic acid [22]. This acid can be a nutraceutical ingredient because it is capable of providing beneficial health effects, including prevention and/or treatment of a disease [23]. Some studies report that it effectively suppresses growth of cancer cells, lowers serum lipid levels in mammals, and has been proposed as chemotherapeutic agent against breast cancer. *P. serotine* seed oil increase its potential as functional and nutraceutical ingredient [22].

Biotic and abiotic metabolites can contaminate crops and plant-based foods; therefore, toxins must be examined [24]. Cyanogenic glycosides occur in a wide range of food plant species, such as cassava root, apples, lima beans, passion fruit, and almonds [25]. Almonds contain amygdalin as a cyanogenic glycoside (a secondary metabolite) [26]. This metabolite produces hydrogen cyanide (HCN) when it is hydrolyzed. Its effects go from intoxication symptoms to neuropathic problems [27]. Nevertheless, the toasting process to which *P. serotine* seed is subjected as snack, helps to not produce amygdalin because of the temperature it is subjected to. The pericarp of *P. serotine* accumulates amygdalin, but it is acyanogenic because it lacks enzymes to release HCN [28]. In addition, it is devoid of oil content, as well as cyanide components [22]. All these circumstances, along with the fact that the seed has been used for human nutrition since ancient times, allow us to assume that it has little or no toxicity [28].

However, there are some treatments that can reduce or eliminate the risk of poisoning, whereby the focus in on removal of glycoside through washing and/or pressing the food, by enzymatic breakdown of the glycoside, destroying the enzyme or a combination of these methods [29].

From *P. serotine* seed, two valuable products can be obtained, namely α-eleostearic acid with nutraceutical potential applications and the defatted seed with high protein content, which can be used for the development of biscuit products and concentrate protein for the stabilization of emulsions.

We have previously evaluated the study of *P. serotine* oil, so we are focusing on the second product and its derivates. Therefore, the aim was to evaluate *P. serotine* defatted flour without hydrogen cyanide risk in cookies and protein concentrate in emulsion stability.

## 2. Materials and Methods

*P. serotine* seeds were obtained from Xochimilco’s market in Mexico City, Mexico. Wheat flour (*Triticum* spp.) and canola oil were purchased from a local food store in Monterrey, Nuevo Leon, Mexico. Whey protein concentrate (WPC, MB Pro-mix, 80%) was food grade from Marquez Bros, International, Inc.-whey division, Hanford, CA. Solvents: hexane, *n*-propanol, boric acid, ethanol, phosphoric acid, and hydrochloric acid were of analytical grade (J.T. Baker reagents, Azcapotzalco, Mexico City, Mexico). The reagents sodium chloride, sodium hydroxide, sodium azide, sodium carbonate, sodium tartrate and copper sulphate were purchased from Development of Chemical Specialities in Monterrey, Nuevo Leon, Mexico. Picric acid was from Acce Microbiology in Guadalupe, Nuevo Leon, Mexico, and Coomassie brilliant blue G-250 from ThermoFisher Scientific, Mexico. Sucrose, Tris(hydroxymethyl)aminomethane, SDS, Folin & Ciocalteu’s and bovine serum albumin (BSA) were from Sigma-Aldrich, Mexico.

### 2.1. P. serotine Defatted Flour

**Scheme 1:**
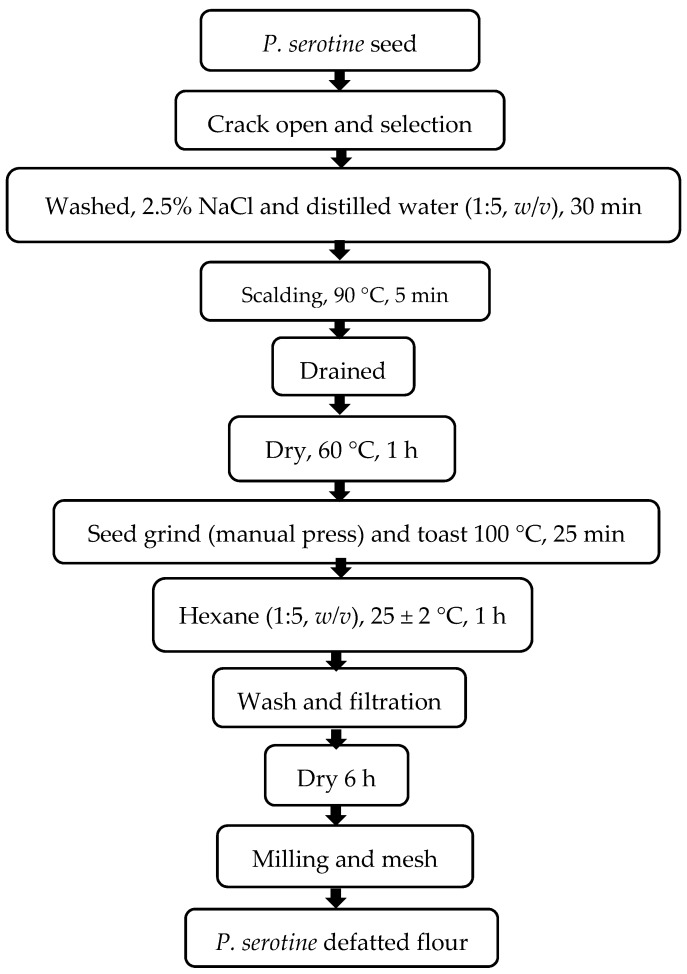
Physical-chemistry process to obtain *P. serotine* defatted flour.

A defatted flour was elaborated from the seeds of *P. serotine* (Scheme 1). Seeds were cracked open with a sterilized metal squeezer, washed with 2.5% NaCl and distilled water (1:5, *w/v*) for 30 min with constant magnetic stirring, followed by scalding with hot water at 90 °C for 5 min, and drained for 7 min, followed by drying for 1 h at 60 °C in an oven with air circulation. Once dried, the oil was removed with a manual oil press (Kinetic, Henan Wecare Industry Co. Ltd., Jiaozuo, China) and the residue (ground seed) toasted to 100 °C for 25 min [29]. Subsequently, the remaining oil was removed by constant magnetic stirring with hexane (1: 5, *w/v*) to 25 ± 2 °C for 1 h. The ground seed was washed and filtered through Whatman paper No. 4 and dried in a hood extractor for 6 h. Finally, it was chopped in a blender and passed through a 70-mesh screen to obtain a *P. serotine* defatted flour [2].

The flour yield was determined by the following formula:

(1) $$Flour\;yield\;\left(\%\right)=\frac{Flour\;weight\;\left(g\right)}{Seed\;weight\;\left(g\right)}*\;100$$

### 2.2. Particle Size

In order to measure the particle size of the *P. serotine* defatted flour, the methodology of Khor et al. [30] was used with some modifications. The flour was measured in a Mastersizer 3000 Hydro LV (Malvern Instruments Ltd., Worcestershire, UK) using the liquid unit. The particle size was evaluated through the volume-weight mean diameter (D_4,3_) at 25 ± 2 °C, as Belorio et al. [31] report. Optical properties of the sample were defined as refractive index 1.37, isopropyl alcohol as dispersant, and an absorption of 0.1. The results were expressed in µm as means ± standard deviation. Wheat flour (*Triticum* spp.) was used as control.

### 2.3. Chemical Composition

Analysis were performed on *P. serotine* defatted flour by using Association of Official Analytical Chemistry [32] and compared with wheat flour (control). Moisture, ash, and crude fiber were evaluated gravimetrically (AOAC 14.006, AOAC 925.15 and AOAC 962.09, respectively). The Goldfish method (AOAC 920.36C) was used to determine the fat content. The protein was measured using the Kjeldahl method (AOAC 930.29) and total carbohydrates were determined by the difference using the following equation:

(2) HC (%)=[100−(protein+lipids+ash+crude fiber)]

### 2.4. Grignard Test

To verify that during the process of making *P. serotine* defatted flour, hydrocyanic acid (HCN) was eliminated, a qualitative test was used according to Castro and Rodriguez [33]. Picrosodic papers were prepared and then circles of filter paper (Whatman No. 4) were soaked with 1% picric acid solution and allowed to dry in the dark until they changed color to deep yellow. Once dried, they were impregnated with 10% sodium carbonate and allowed to dry. Afterwards, they were fixed on the lid of amber bottles and two drops of 10% sodium carbonate were added, preventing it from dripping.

The *P. serotine* defatted flour was placed inside the jar to fill a third of it and covered quickly. The bottles were stored in the dark and after 24 h, a reading was taken. As a control, only fractionated *P. serotine* seeds were used (without any treatment). If the paper´s yellow color was maintained or it became light orange, there would be absolutely no problem in its consumption, but if it changed to intense orange or pink, it could only be consumed with caution and if the color became reddish or dark brown, it would not be safe to consume.

### 2.5. Cookie Preparation

Four variations of cookie recipe were made according to Jia et al. [34] with some modifications. The cookie dough formula is presented in Table 1. The control recipe was 100% commercial wheat flour (Fc) and the other four were 100, 75, 50, and 25%, respectively, with *P. serotine* defatted flour (F1 to F4, respectively). Butter and sugar were mixed, then creamed with a Kitchen Aid mixer at low speed for one min. Vanilla essence and egg were added and mixed for one minute. In another bowl, all dry ingredients (flour, baking powder, and salt) were sifted and gradually added to the previous mix at low speed for 1 min and then medium speed for one min. When all the ingredients were integrated and homogenized, the dough was wrapped in plastic and allowed to cool at 4 °C for 1 h.

Once the dough had rested, it was kneaded and spread with a rolling pin and 2 × 2 cm and 0.5 cm high square cookies were cut and, placed in an aluminum tray with waxed paper to prevent them from sticking. The oven was preheated at 160 °C for 15 min and the cookies were baked for 12 min at the same temperature. After removal from the oven, the cookies were left to cool at room temperature (25 ± 2 °C).

### 2.6. Sensory Evaluation and Chemical Composition

The evaluation was carried out in the Sensory Evaluation Laboratory of the Faculty of the College of Food Science at the Autonomous University of Nuevo Leon, Mexico. Fifty-five panelists (untrained) participated in the sensory test based on Jia et al. [34]. These individuals were seated at individual tables in different compartments. A 9-point hedonic scale was used (1 = extreme dislike, 5 = neither like nor dislike, 9 = extreme like) to evaluate the cookies texture, appearance, color, smell, taste, mouthfeel, aftertaste, and overall impression. Scores of five and higher for overall impressions were considered acceptable in this study. Cookies with 3-digit random number codes were randomly presented to the panelists, who were instructed to cleanse their palates with distilled water (25 °C) between sensory analyses. Chemical composition analysis (fat, protein, crude fiber and carbohydrate) involved quantification in the cookies with the highest score for the overall impression attribute, as specified in Section 2.3.

### 2.7. Extraction of Soluble Proteins

Proteins were extracted sequentially from *P. serotine* defatted flour according to the procedure described by Ramirez Pimentel et al. [35] and Raya Perez et al. [28], with the following solvents: distilled water (albumins), 0.5 M NaCl solution in 50 mM Tris pH 8 (globulins), 55% (*v/v*) 2-propanol (prolamins) and 0.1 M boric acid with 0.5% SDS pH 8 (glutelins).

The flour:solvent mixture (ratio 1:10, *w/v*) was stirred for 1 h at 25 ± 2 °C. The extracts were centrifuged (Hermle Z326, Labortechnik GmbH, Wehingen, Germany) at 13,000 *g* at 25 ± 2 °C for 20 min and the supernatants filtered (Whatman No. 4). The extraction with each solvent was repeated on the same sample sequentially and the supernatants of the three extractions were combined.

### 2.8. Soluble Protein Determination

Soluble proteins were quantified from the soluble protein extractions as reported by Lopez Dellamary Toral [36] based on the Bradford [37] technique, with some modifications. Bovine serum albumin (BSA) was used as a standard (0.05 to 0.5 mg/mL). The soluble protein fractions were diluted with 50 mM Tris-HCl buffer at pH 7, to obtain values within the standard range concentration. Albumin concentration was 0.49 mg/mL, globulin 0.26 mg/mL, prolamin 0.33 mg/mL, and glutelin 0.24 mg/mL. In microplates, 20 µL of each extract was added in triplicate, using wells consecutively with 200 µL of Bradford reagent (0.01% Coomassie Blue G-250, 4.75% ethanol, 85% H_3_PO_4_), allowing to stand for 2 min. The samples were evaluated (microplate reader-Anthos 2020 version 2.0.5) at 620 nm.

### 2.9. Electrophoresis

Protein patterns were analyzed according to Syros et al. [38] with some modifications based on Bio-Rad laboratories [39] using polyacrylamide gel electrophoresis (SDS-PAGE). Two glass plates were placed in the electrophoresis chamber, fixing them with plastic spacers and polyacrylamide gel (4–20%) with a 10-well comb (Mini-PROTEAN TGX, Precast protein gels, Bio-Rad Laboratories, Inc. Irvine, CA, USA).

In gel rails, 20 µL of each extraction of soluble protein fraction were placed (at the same previous concentrations) with distilled water. After electrophoresis, the gel was completely immersed in a fixing solution and washed three times for 10 min with distilled water. The gel was immersed and stirred in Coomassie blue dye solution (G-250) until bands were clearly evidenced.

### 2.10. Isoelectric Point (pI)

The isoelectric point of the *P. serotine* defatted flour was determined according to the theoretical determination of proteins and other macromolecules, through zeta potential (ζ-potential) which is the most direct characterization of the repulsion or attraction strength between their acid-base residues [40,41]. For this, a mixture of flour: deionized water in a 1:20 ratio (*w/v*) at different pH with 0.1 N NaOH and 0.1 N HCl was vortexed for two minutes. Zetasizer Nano ZS90 light scattering equipment (Malvern Instruments, Worcestershire, England, UK) was used. The measures were in automatic mode using a universal immersion cell (ZEN 1002, Malvern Instrument, Worcestershire, UK) at 25 °C. The results were reported as the average of three separate injections, with three measures per injection. The averages of triplicate values were used as the values for zeta potential reported.

### 2.11. Prunus serotine Protein Concentration (PsPC)

To obtain *Ps*PC the results obtained from the p*I* were taken as a basis. Variations of the procedure were undertaken to determine the one that was repeatable and had protein concentrate values ≥ 80% and ≤ 90%. All procedures were initiated by mixing the *P. serotine* defatted flour for 1 h in vortex with distilled water at pH 11 with 0.1 N NaOH (25 ± 2 °C), ratio 1:20 (*w/v*). Then the sample was isolated by centrifugation (Hermle Z326, Labortechnik GmbH, Wehingen, Germany) at 13,000 *g* for 30 min and filtered through No. 4 Whatman paper to obtain two fractions (residue and supernatant).

In the first variation, up to two extractions of the residue obtained in the first part of the process were carried out with 5% NaCl (1:20, *w/v*), at two extraction times (30 min and 1 h) in vortex at 25 °C, followed by centrifugation (Hermle Z326, Labortechnik GmbH, Wehingen, Germany) at 13,000 *g* for 30 min and filtered, again obtaining two fractions. The residue was analyzed utilizing the Kjeldhal method [32] to ensure the lowest protein loss in the process. The resulting supernatant was combined with the supernatant obtained in the first part, to subsequently acidify and solubilize the protein with HCl as shown in Scheme 1. The precipitate was stored until analysis at −20 °C. The pH for acidification were 3.0, 3.7, and 4.5.

**Scheme 2:**
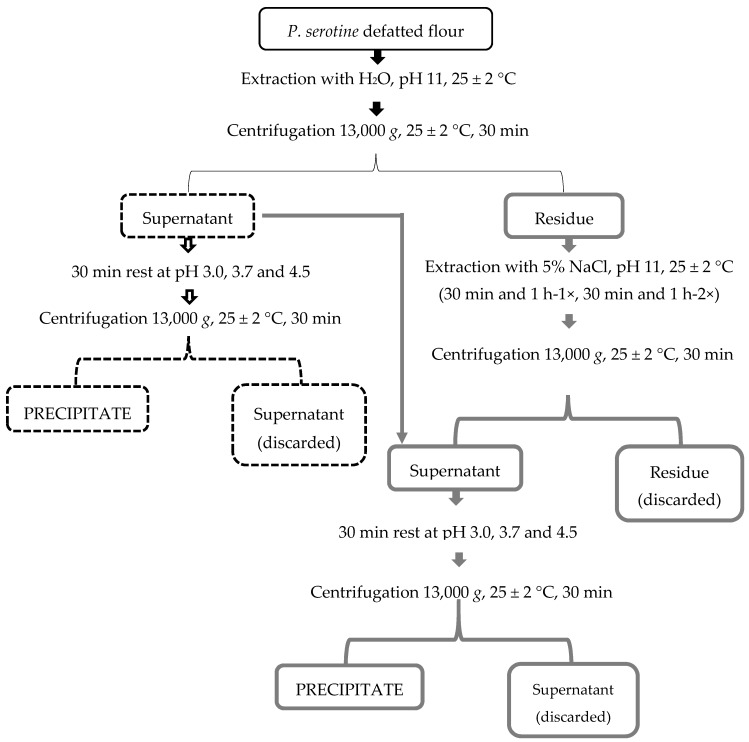
The diagram in gray indicates the procedure for obtaining protein concentrate from the *P. serotine* defatted flour with NaCl treatment and acid pH. 1× indicating that the extraction was performed once, and 2×, that it was repeated twice. The black dots indicate the procedure for obtaining protein concentrate from the *P. serotine* defatted flour by direct acidification.

In the second variation, the residue of the first part was automatically discarded and the supernatant was acidified with HCl and left to rest for 30 min. Finally, it was centrifuged and filtered under the previous conditions. The precipitate was collected and stored at −20 °C until use. Three acid pH values (3.0, 3.7, and 4.5) were tested (Scheme 2).

All final precipitates were analyzed according to proximal analysis via the Kjeldhal method based on AOAC 930.29 [32]. The yield was determined by the following equation:

(3) $$Protein\;concentrate\;yield\;\left(\%\right)=\frac{Precipitate\;weight\;\left(g\right)}{Defatted\;flour\;weight\;\left(g\right)}*\;100$$

### 2.12. Preparation of Emulsions

The emulsifying agents (*Ps*PC and whey protein concentrate) were prepared at 1% *w/v* in deionized water and solubilized with constant stirring. Then the sample was allowed to hydrate overnight at 4 °C. Afterwards, 0.05% sodium azide was mixed to prevent microbial growth. Different amounts of canola oil 0.1, 1, 5, 10, and 20 g were added, to obtain a variety of mass fractions (ϕ = 0.002, 0.02, 0.1, 0.2, and 0.4). The emulsions were mixed in a homogenizer (OMNI International GLH, Georgia, United States) at an initial speed of 1000 rpm for 2 min and subsequently at 3000 rpm for 3 min. All emulsions were made in triplicate and stored at 25 ± 2 °C for 18 days, and every three days, all the following analyzes were made. Whey protein concentrate (WPC) was used as a control [42,43].

### 2.13. Droplet Size Measurement

Particle size was determined by integrated light scattering using a Mastersizer 3000 Hydro LV (Malvern Instruments Ltd., Worcestershire, UK). The emulsions were analyzed immediately after preparation in quintuplicate. Laser diffraction measures the particle size distribution (diameter equivalent to the volume) from the angular variation of the intensity of scattered light when the laser beam passes through the particles dispersed in solution. The data are then integrated based on the angular dispersion intensity, calculating the particle size through the Mie theory of light scattering. The droplet size of emulsions was evaluated through volume-surface mean diameter (D_3,2_) at 25 ± 2 °C as Guo and Mu reports [1]. Optical properties of the sample were defined as refractive index 1.43 for *Ps*PC and 1.46 for WPC, water as dispersant and an absorption of 0.1. The results were expressed as means ± standard deviation [30].

### 2.14. Emulsifying Activity Index and Emulsifying Stability Index (EAI and ESI)

The EAI and ESI were assayed via the colorimetric method, previously reported by Guo and Mu [1]. Immediately after homogenizing each emulsion, 20 μL from the bottom was taken and diluted with 5 mL of 0.1% SDS solution. It was vortexed for 5 min and, the absorbance was measured in a spectrophotometer (UV-Visible-Genesys 10s, Thermo scientific, Cambridge, MA, USA) at 500 nm [1]. The *EAI* and *ESI* were calculated using the following equations:

(4) EAI (m2g)=2 ∗ 2.303 ∗ A0 ∗ dilution factorc ∗ 1 ∗ (1−ϕ) ∗ 10,000

where *c* is the initial protein concentration which is 1% *w/v*, ϕ is the oil weight fraction, dilution factor was 250.

(5) $$ESI\;\left(\min\right)=\frac{A_{0}}{A_{0}-A_{10}}*\;t$$

where *A*_0_ and *A*_10_ are the absorbance of the diluted emulsions at 0 and 10 min, respectively and, *t* was 10 min.

### 2.15. Interfacial Protein Concentration

According to Eichberg and Mokrasch [44] and Guo and Mu [1], interfacial protein concentration was quantified. Two milliliters of freshly prepared emulsions were diluted with 2 mL of 50% sucrose solution (*w/v*) and vortexed for 5 min at 25 ± 2 °C. In a centrifuge tube, 2 mL of the solution were mixed with 7 mL of 5% sucrose solution (*w/v*) and all samples were centrifuged (Spectrafuge 6C, Labnet International, Inc., New York, NY, USA) at 3500*g* for 30 min at 25 ± 2 °C. Once centrifuged, three phases were observed: the oil drops in the upper phase, an intermediate phase corresponding to the 5% sucrose solution, and the aqueous phase in the lower part of the tube. The tubes were frozen at −40 °C for 24 h and then the upper layer of the oil was removed.

The proteins adsorbed from the oil phase were removed by adding 20 mL of 1% SDS (*w/v*) solution. To determine the concentration of adsorbed protein, 1 mL of the sample was mixed with 3 mL of an alkaline copper reagent (A: 2% Na_2_CO_3_, 0.4% NaOH, 0.16% sodium tartrate, and 1% SDS with B: 4% CuSO_4_. 5H_2_O, in a ratio of 100:1). The samples were vigorously stirred, and allowed to rest at 25 ± 2 °C for 10 min. Subsequently, 0.3 mL of 2 N Folin-Ciocaletu was added and allowed to stand for 45 min at 25 ± 2 °C. The absorbance was immediately measured at 660 nm in a spectrophotometer (UV-Visible-Genesys 10s, Thermo scientific, Cambridge, MA, USA) against a blank. Bovine serum albumin (BSA) was used as a standard. The interfacial protein concentration was calculated as:

(6) T (mgm2)=Cad/SV

where *C_ad_* (mg/mL) is the concentration of adsorbed protein and *S_V_* is the specific interfacial area (m^2^/mL emulsion) of the emulsion droplets.

### 2.16. Optical Microscopy

The optical microscopy photographs were taken based on Huang et al. [45] with small modifications. Emulsions were mixed in vortex for 1 min prior to analysis. A drop of the emulsion was placed between the coverslip and microscope slide. The globules of the emulsions were examined and observed under bright field illumination with 40× objective lens on a Leica microscope (Leica DM500, 9435 Heerbrugg, Switzerland) along with the software Leica LAS EZ 2.0.0, Ltd., Application Suite (Leica Microsystems, 9435 Heerbrugg, Switzerland).

### 2.17. Statistical Analysis

Data from the replicated experiments were analyzed to determine whether the variances were statistically homogeneous, and the results were expressed as the mean ± standard deviation (SD). Statistical comparisons were made by one-way variance analysis (ANOVA) followed by Tukey’s test using Statgraphics centurion XVII Software. The difference between means was considered significant at *p* < 0.05.

## 3. Results and Discussion

### 3.1. Particle Size of Defatted Flour

*P. serotine* defatted flour had a yield of 71.07 ± 2.10%. Its particle size (D_4,3_) was 5.10 ± 0.03 µm, which was minor for the commercial wheat flour (*Triticum* spp.) with 7.31 ± 0.01 µm, making *P. serotine* flour 1.4 times smaller.

The AOAC 965.22 [46] mentioned that wheat flour must be able to pass through a No. 70 mesh (212 µm) to be acceptable commercially, and *P. serotine* flour in the process of elaboration did pass through this mesh screen and its particle size was smaller than that of wheat flour (control).

It is already known that the particle size of wheat flour can influence cookie quality, but this can also be true for gluten-free flours. Belorio et al. [31] observed that cookies with smaller values of the elastic component (*G*’) correspond to flours with higher values of D_4,3_. Meanwhile, the biggest elastic values (*G*’) were found in doughs elaborated from finer flours.

### 3.2. Chemical Composition

The moisture in both flours passed the test mentioned in Codex Standard 152-1985 [47]. *P. serotine* defatted flour was 4.6 times less moisture than wheat flour (Table 2). It was not possible to remove all the oil contained in the *P. serotine* seed. After the separation process, 3.4% was quantified. Possibly because of the oil being bound to proteins contained in *P. serotine*, it also contains 5 times more protein and 1.5 times as much total fiber than wheat flour and 2 times less carbohydrates [48].

### 3.3. Grignard Test

Zumaeta Cordova and Gonzales Díaz [29] mentioned that the treatments that allow the release of hydrocyanic acid from glycosides and their subsequent elimination by drying or heating, are those that guarantee greater safety (100 °C for 25 min).

**Figure 1:**
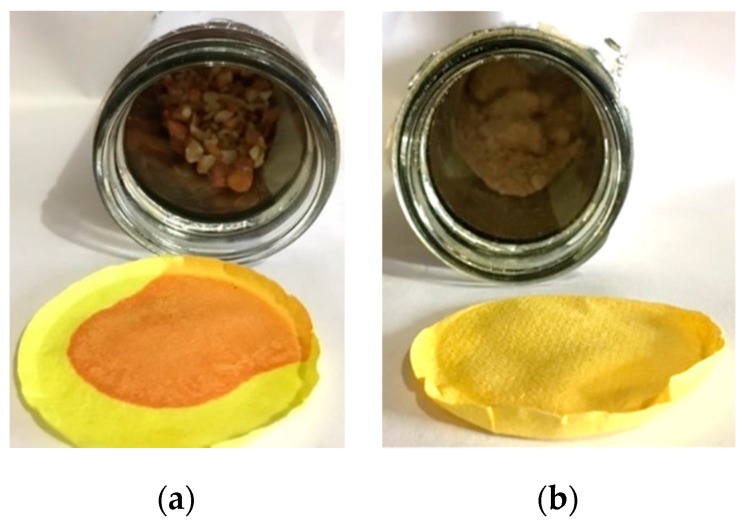
(**a**) *P. serotine* seed without treatment. (**b**) *P. serotine* defatted flour with treatment.

The experiment showed that in the control sample (fractionated seed without treatment), the paper impregnated with picric acid changed from yellow to deep orange, which indicated that the food could be consumed, but with caution. Meanwhile, the flour with treatment paper remained with the same yellow color. This indicated that there were no potential consumption problems (Figure 1).

We believe that due to the low concentration of cyanogenic compounds, the consumption of *P. serotine* seeds has not caused any poisoning problems. In addition, the seed has been toasted for a long period of time prior to consumption.

According to Alveano Aguerrebere [20], there is no significant difference between the protein content of the seed in its toasted version (37.95 ± 0.16%), compared to when it is raw (36.55 ± 0.22%). Also, Garcia Aguilar et al. [18] mentioned that there is no significant difference in the values of in vitro protein digestibility of raw (88.12 ± 0.72%) and toasted (89.40 ± 1.32%) *P. serotine* seeds.

### 3.4. Sensory Evaluation

The effects of the addition of *P. serotine* defatted flour are shown in Table 3. A decrease in the scores of all sensory attributes and the overall impression of the cookies was found with the addition of *P. serotine* defatted flour. However, the maximum amount of *P. serotine* defatted flour accepted by the untrained panelists in the cookies was 75% (F2) based on the value obtained in the overall acceptability category though the formulation with *P. serotine* defatted flour showing the highest acceptability level was F3 with a substitution of 50%.

The results obtained in the evaluation show that the cookies that were made with 75 to 25% *P. serotine* defatted flour (F2 to F4) received a score greater than five in overall impression, making them acceptable. These cookies had greater acceptance compared to other cookies made with Californian almonds (*P. dulcis*), for which maximum acceptance was only 20% substitution, with a score of 5.25 in overall impression [34].

### 3.5. Cookie Chemical Composition

Based on the results obtained from the sensory test, a decision was made to carry out the chemical analysis on Fc cookies (100% wheat flour) because it was preferred by the evaluators. Of the cookies made with *P. serotine* defatted flour, those substituted by 50 and 25% (F3 and F4) were selected as a result of obtaining the highest score in overall impression (Table 4).

Cookies made with *P. serotine* defatted flour stood out for having fiber and for their protein content, up to 17 times higher than cookies with wheat flour (Fc), as well as, for presenting 6.57 and 2.35% lower carbohydrates, and 6.14 and 9.6% lower fat content (F3 and F4, respectively) than control cookies. In addition, it can be said that cookies made with 50 and 25% *P. serotine* defatted flour have a lower gluten content compared to control cookies, since almonds are the best vegetable sources of gluten-free protein and one of the most popular ingredients in the preparation of gluten-free foods, making them a healthy alternative for people suffering from celiac disease [49].

### 3.6. Soluble Protein Determination

From the *P. serotine* defatted flour 16.4 ± 2.54 g soluble protein was extracted/100 mL of solution, which is equivalent to 32.15 ± 0.49% of total protein content. The soluble protein profile was albumin 76.95 ± 2.29%, globulin 13.60 ± 2.56%, glutelin 6.16 ± 0.99%, and prolamin 3.29 ± 0.37%. The relative concentration of soluble protein with respect to insoluble proteins was 3.3:1 (Table 5). Raya Perez et al. [28], also quantified soluble protein in *P. serotine* and also reported albumin as the predominating fraction, followed by globulin, glutelin, and finally prolamin.

### 3.7. Electrophoresis

**Figure 2:**
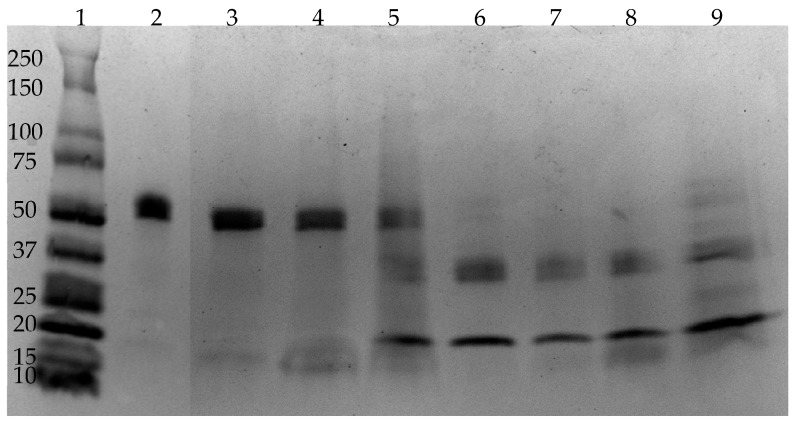
Soluble protein patterns of *P. serotine* defatted flour. 1. Molecular weight markers, lanes 2 to 5 (without 2-mercaptoethanol); 2. albumin; 3. globulin; 4. prolamin; 5. glutelin. Lanes 6 to 9, as lanes 2 to 5 but with 2-mercaptoethanol.

The SDS-PAGE patterns for *P. serotine* defatted flour is reported in Figure 2. The molecular weight of albumin varied in a range from 63 to 20 KDa (lane 2 and 6). In globulins, it varied between 63 and 20 KDa (lane 3 and 7), in prolamins, it ranged from 60 to 20 KDa (lane 4 and 8), and in glutelins from 60 to 12 KDa (lane 5 and 9).

The molecular weights obtained were similar to the ones reported by Raya Perez et al. [28]. Albumin weight varied between 65 and 20 KDa, globulin between 65 and 14 KDa and prolamins and glutelins between 65 and 12 KDa, respectively.

Albumins and globulins are the main storage proteins of dicotyledonous plants (e.g., legumes and oilseeds), whereas prolamins and glutelins predominate in monocotyledonous plants (e.g., cereals). As expected of a nitrogen source, storage proteins are rich in asparagine (and aspartate), glutamine (and glutamate), and arginine [50], which is the case of *P. serotine* seed. According to Garcia Aguilar et al. [18] the seed contains 116.97 mg/g of asparagine, 273.73 mg/g of glutamine, and 87.42 mg/g of arginine (toasted version), the three amino acids showing the highest values.

Sze Tao and Sathe [2] have reported that pepsin is the most efficient protease hydrolyzing almond (*P. dulcis*) protein, especially for polypeptides with molecular weights from 15 to 42 KDa. Typically, pepsin hydrolysis produced polypeptides with 15 to 36 KDa, followed by 15 to 20 KDa and some with 20 to 40 KDa. Therefore, *P. serotine* defatted flour protein may be useful in production of food protein hydrolysate and did not necessarily involve an additional process.

### 3.8. Isoelectric Point (pI)

The isoelectric point of an amino acid is the pH value at which the amino acid is doubly ionized or in zwitterion concentration and is deduced from the Henderson–Hasselbach equation, as the average of the p*K* values of the stages that form and decompose the zwitterion. The point of intersection of calibration curve with the x-axis is p*I* value of protein [34,51].

**Figure 3:**
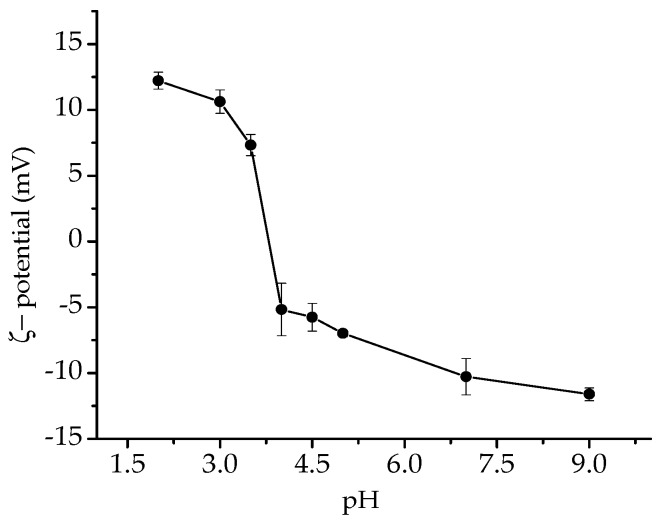
Isoelectric point (p*I*) of *P. serotine* defatted flour as a function change of concentration of hydrogen concentration.

As a result of the conductivity measurement at different pH of the *P. serotine* defatted flour, it was found that the specific p*I* for this oilseed was 3.7 (Figure 3). This value can be attributed to the high content of acidic amino acids present in the oilseed (aspartic acid 112.29 mg/ g and glutamine 256.84 mg/g), which influenced the low value of p*I* [17]. In addition, this value is within the optimum range for the precipitation of oil proteins such as peanuts (4.0 ± 0.25), coinciding with what other researchers have reported [52].

### 3.9. Prunus serotine Protein Concentration (PsPC)

The *P. serotine* defatted flour was subjected to different treatments to obtain a process that is repeatable, standardized, and therefore reliable. The processes were adjusted and carried out as the results were obtained.

Usually to solubilize protein from oilseed meal, alkaline solutions are used. Solutions with a pH of 9 to 12 have higher protein yields. However, in values of pH 12 and higher, isolates with better quality are not always obtained [53].

The salts increase the solubility through the salting-in process, whereby the counter ions cover the ionic charges of protein molecules [54]. NaCl is a solubilizing agent, and the combination of alkali and salt is often used to improve protein solubility [53]. Table 6 shows the results of treating the flour with an alkaline pH followed by the interaction with a saline solution at different numbers of extractions in order to extract and recover the highest protein content of the first residue of flour.

The sample subjected to two extractions of one hour each with NaCl at a 1:20 (*w/v*) ratio at pH 11 had the lowest remaining protein in the residue (13.30 ± 0.39%), which would indicate that this process allows the collection of more protein in the supernatant of the treated sample.

The most common approach to recover solubilized proteins is by precipitating it with pH adjustment. In peanuts, some authors use pH 4.5 [53,54], while other researchers mention that the optimum pH can be within the isoelectric region between pH 3.0 and 5.0 [47]. Based on all these data, the following pH were used to precipitate the proteins of the supernatants: 4.5, 3.7, and 3.0.

The final protein content precipitated from the collected supernatant was not enough to reach the desired value of protein concentrate (whey protein concentrate ≥ 80%). The resulting values were: 56.77 ± 9.20 (pH 4.5), 66.96 ± 1.21 (pH 3.7), and 72.02 ± 5.01 (pH 3.0). This was attributed to the fact that at a high ionic strength, proteins can be almost completely precipitated from their solution because of dehydration in the protein molecules, thus reducing their solubility [53]. Therefore, the number and time of extraction was reduced in this, as well as in the flour:NaCl ratio (Table 6). At the same time, tests were carried out on the *P. serotine* defatted flour involving interaction only with an alkaline solution at pH 11 and then the supernatant was acidified to identify, in which acidic pH was the most effective.

The results obtained when precipitating the supernatants at pH 3.0 compared to other pH values (3.7 and 4.5) yielded a higher protein percentage. This was corroborated with the direct acidification process, which showed a concentrate value of 81.99 ± 6.96%.

The procedure was repeated two more times and results show that the treatment with direct acidification was the most effective. The average value of protein concentration in the final *P. serotine* defatted flour precipitate was 82.0%.

Researchers have identified and quantified the amino acids present in *P. serotine* seed, as well as its total and soluble protein [18,20,40]. However, there are still no reports on the uses or applications of *P. serotine* protein concentrate, making this work one of the first in its findings.

### 3.10. Droplet Size Measurement of Emulsions

**Figure 4:**
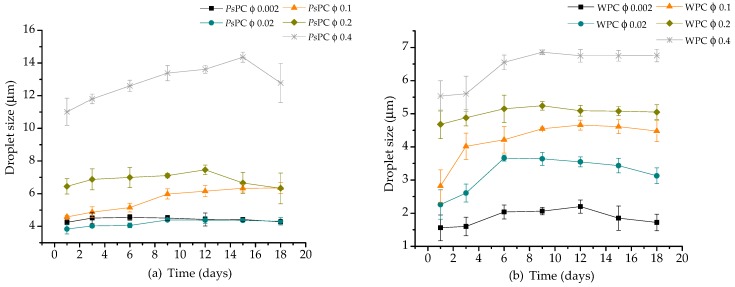
Droplet size (D_3,2_) of emulsions with (**a**) protein concentrate (*Ps*PC) (1% *w/v*) compared to (**b**) whey protein concentrate (WPC) (1% *w/v*) at different ϕ.

In Figure 4, it can be seen that emulsions with more oil content had the largest droplet size. As the days passed, the particle size increased when ϕ ≥ 0.2. In every *Ps*PC emulsion, the maximum droplet size value was reached at different days, but it could be considered as average on day 10. While in WPC emulsions, the range of days to reach the maximum droplet size was more stable (between days 6 and 12), the particle size was more dispersed compared to the *P. serotine* protein.

The *Ps*PC emulsion at ϕ 0.02 presented a droplet size of 4.39 ± 0.08 µm as a maximum, becoming the smallest and more constant emulsion during the time of experiment, and for WPC, it was at ϕ 0.002, with a value of 2.20 ± 0.20 µm. In both the control (WPC) and *Ps*PC emulsions, on the other hand, at ϕ 0.4, the droplet size had the highest value of 6.88 ± 0.11 µm (day 9) and 14.36 ± 0.31 µm (day 15), respectively, during storage time because of coalescence.

A small droplet size is of interest in emulsion studies, because they are strongly correlated with high emulsion stability [16]. Authors such as Pandolfe [55] and Floury et al. [56] have reported that the increase of oil in emulsions led to a gradual increase of oil droplet sizes. Part of the effect may be due to the limitation of surfactants in emulsions, since as the oil content increases, the available proteins decreases, limiting the stabilizing benefits of the protein, thus favoring the coalescence of the oil drops, and therefore, increasing the diameter. We suggest that emulsion stability is due to the hydrophobicity of the polypeptide chain. The mean diameter of the droplets in food emulsions can vary from less than 0.2 µm (for cream liqueurs) to greater than 100 µm (for salad dressings), depending on the product [57].

### 3.11. Emulsifying Activity Index and Emulsifying Stability Index (EAI and ESI)

**Figure 5:**
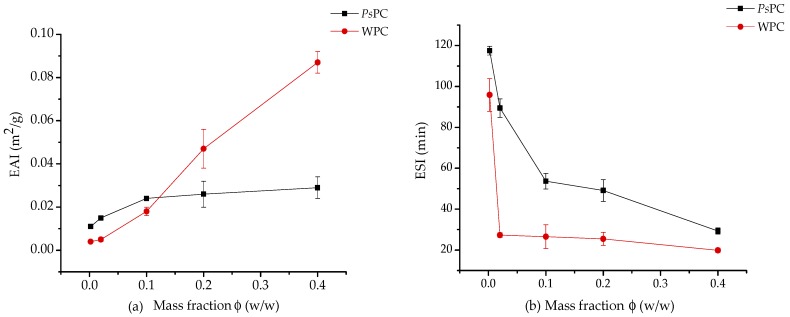
(**a**) Emulsifying activity index (EAI) and (**b**) emulsifying stability index (ESI) of *Ps*PC emulsions compared with WPC at different ϕ.

Compared with other particles, the protein particles have emulsifying properties and great potential to form soft particles [42]. The ability of a protein to form an emulsion can be defined as an emulsifying activity index (EAI), which determines the approximate amount of interfacial area that can be stabilized per unit amount of protein. Additionally, the stability of the emulsion over a specific time period is referred to as the emulsifying stability index (ESI) [58]. The EAI increased as did the mass fraction in *Ps*PC emulsions (Figure 5). The effect was similar in the control emulsions.

On the contrary, the stability time diminished as the mass fraction (ϕ) increased in ESI (Figure 5). In *Ps*PC emulsions, ESI went from 117.50 ± 2.17 (ϕ = 0.002) to 29.34 ± 1.48 min (ϕ = 0.4) and in WPC emulsions, from 95.83 ± 7.95 (ϕ = 0.002) to 19.87 ± 1.08 min (ϕ = 0.4). For the control emulsions, less stability time was always reported compared to those of *Ps*PC.

Different authors have mentioned similar characteristics in almond proteins, wheat gluten, and acidic subunits of soy (11S globulin) [2,59]. Guo and Mu [1] also got similar results when they studied emulsifying properties of sweet potato protein and found that at low protein concentrations (<1%, *w/v*), the EAI values are greater, because it facilitates the formation of new droplets, and with the increase of oil, ESI value decreases. Nevertheless, at oil volumes >35% *v/v*, there is a marked increase in ESI, a phenomenon that has been reported also by Sun and Gunasekaran [60] for whey protein isolates.

EAI can be related with interfacial effect and low interaction with aqueous solutions. Our previous droplet size results can be associated with the EAI; as these indexes increased, the droplet size also increased. This can be attributed to the proteins that are surface active molecules with the capacity to improve the stability of oil-in-water emulsions, creating a protective membrane that generate repulsive interactions between oil droplets [16].

### 3.12. Interfacial Protein Concentration

The effect of interfacial protein concentration shows the oil drops phase separation after being centrifuged. The values suggest that emulsions were stable. When values of ϕ < 0.2 were used, it was difficult to separate the phases and reported the values.

Table 7 shows the results of emulsions with ϕ 0.2 and 0.4. Control emulsions with WPC showed that as the volume of oil increases, the protein content at the interface is diminished.

The interfacial protein concentration in control emulsions was more constant, in contrast with *Ps*PC, which showed a higher value as oil volume increased, allowing more stability.

### 3.13. Optical Microscopy

**Figure 6:**
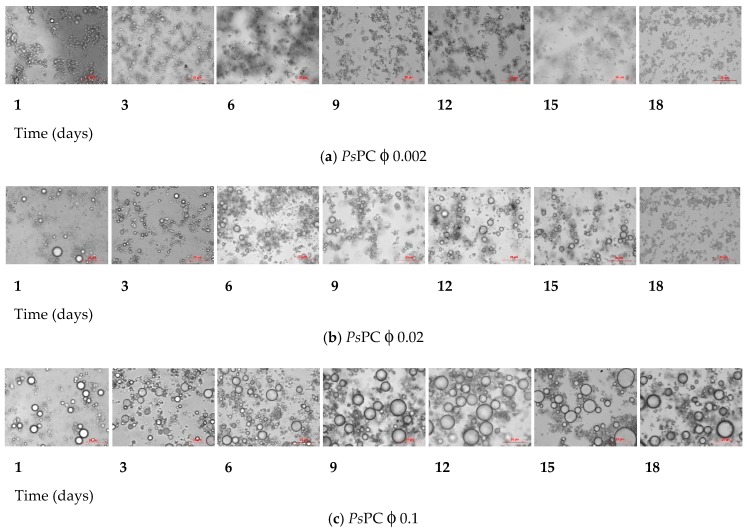

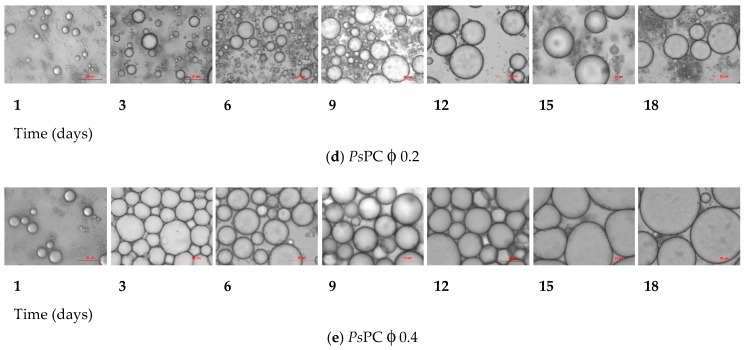
(**a**–**e**) Microphotographs of *Ps*PC emulsions at 40×. Microphotographs show particle size in function of oil in aqueous phase.

**Figure 7:**
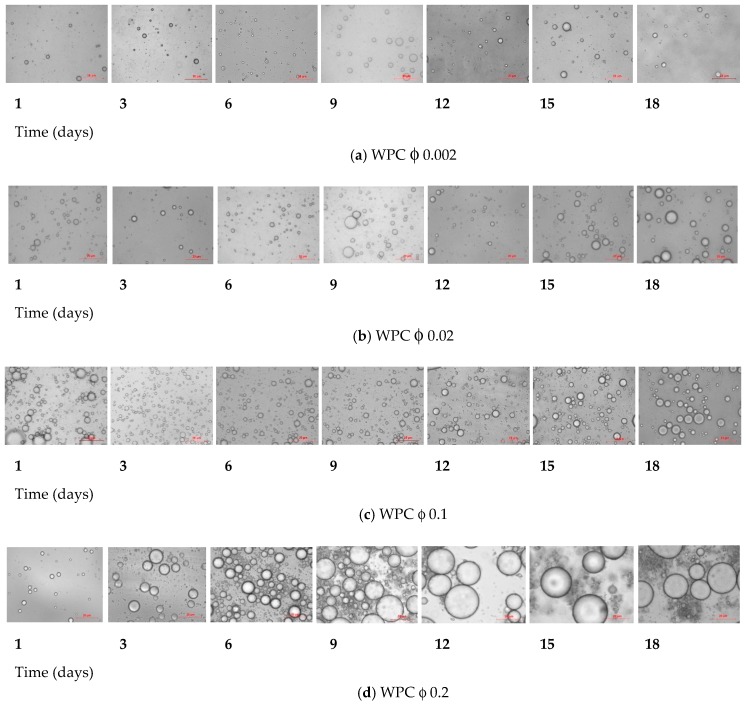

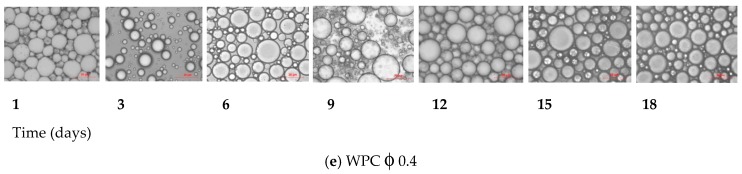
(**a**–**e**) Microphotographs of WPC emulsions at 40×. The particle size changes depending on the oil phase increase.

The microphotographs in function of *Ps*PC and WPC are shown in Figure 6 and Figure 7, respectively. The images reflect the results of droplet size analysis. It shows that as the mass fraction of emulsions increased, the droplet size also decreased and began to show coalescence. Guo and Mu [1] report similar results. The maximum droplet size can be seen between day 9 and 12 in both emulsions.

WPC and *Ps*PC emulsions at their smallest mass fractions (ϕ = 0.002 and ϕ = 0.02) showed more homogenization.

## 4. Conclusions

The *P. serotine* seed is an important source of protein and mainly contains albumin. *P. serotine* defatted flour can be used to replace wheat flour at 50% in the preparation of cookies with acceptable sensory property. We believe that it is necessary to remove all cyanogenic compounds in flour of *P. serotine*. The Grignard test shows a positive reaction in flour without thermic treatment. The content of raw fiber in cookies was almost the same in all treatments. *P. serotine* flour purification can be a concentrated protein source with possible applications to stabilize emulsions.

The alkaline (pH 11) and acid (pH 3.0) process showed a higher concentration of protein than the ionic force process as a function of the sodium chloride concentration. The protein concentrate is comparable to WPC in that it forms stable emulsions with oil content of less than 20% by weight, without changing the particle size during 18 days of storage.

## Figures and Tables

**Table 1:** Cookie dough formula.

Ingredients	Weight (g)
Flour (Fc, F1, F2, F3, F4)	150
Baking powder	3
Salt	3.5
Butter without salt	73
Sugar	66.6
Egg	25
Vanilla essence	2.5

Fc corresponds to 150 g of wheat flour, F1 corresponds to 150 g of *P. serotine* defatted flour, F2 corresponds to 112.5 g of *P. serotine* defatted flour and 37.5 g of wheat flour, F3 corresponds to 75 g of *P. serotine* defatted flour and 75 g of wheat flour and F4 corresponds to 37.5 g of *P. serotine* defatted flour and 112.5 g of wheat flour.

**Table 2:** Nutritional components of two flours.

Component (%)	*P. serotine*	Wheat ^1^ (*Triticum* spp.)
Moisture	2.58 ± 0.09 ^b^	11.92 ^a^
Ash	5.36 ± 0.24 ^a^	0.47 ^b^
Fat	3.36 ± 0.31 ^a^	0.98 ^b^
Protein	50.94 ± 0.64 ^a^	10.33 ^b^
Crude Fiber	4.03 ± 0.27 ^a^	2.7 ^b^
Carbohydrates	36.31 ± 1.09 ^b^	76.31 ^a^

The values are the average of five assays ± standard deviations of the flours. Mean values labeled with a different letter in the same file are significantly different (*p* < 0.005). ^1^ Wheat nutritional values were consulted on https://fdc.nal.usda.gov/fdc-app.html#/food-details/168936/nutrients.

**Table 3:** Effects of *P. serotine* defatted flour on sensory attributes of cookies.

Sensory Attributes	Fc	F1	F2	F3	F4
Color	7.38 ± 2.01 ^a^	4.49 ± 2.01 ^c^	5.93 ± 2.03 ^b^	6.96 ± 1.49 ^a, b^	6.91 ± 1.97 ^a, b^
Smell	7.43 ± 1.78 ^a^	4.81 ± 1.99 ^c^	6.04 ± 2.03 ^b^	6.59 ± 1.46 ^a, b^	6.48 ± 1.77 ^a, b^
Taste	7.38 ± 1.70 ^a^	2.55 ± 1.55 ^c^	5.73 ± 2.18 ^b^	5.53 ± 1.74 ^b^	6.07 ± 2.14 ^b^
Texture	7.33 ±1.88 ^a^	3.95 ± 1.96 ^c^	5.73 ± 1.92 ^b^	5.76 ± 1.73 ^b^	6.65 ± 1.67 ^a, b^
Appearance	7.49 ± 1.99 ^a^	4.56 ± 2.08 ^c^	5.78 ± 2.01 ^b^	6.85 ± 1.42 ^a^	6.78 ± 1.82 ^a, b^
Mouthfeel	7.33 ± 1.76 ^a^	3.16 ± 1.61 ^c^	5.39 ± 2.34 ^b^	5.69 ± 1.76 ^b^	6.05 ± 2.04 ^b^
Aftertaste	6.89 ± 1.72 ^a^	2.89 ± 1.71 ^c^	5.27 ± 2.16 ^b^	5.60 ± 2.12 ^b^	5.78 ± 2.15 ^b^
Overall impression	7.45 ± 1.54 ^a^	3.27 ± 1.72 ^c^	5.84 ± 1.79 ^b^	6.31 ± 1.60 ^b^	6.18 ± 1.88 ^b^

The values are the average of fifty-five assay ± standard deviations of the sensory attributes of cookies with *P. serotine* defatted flour and wheat flour. Mean values labeled with a different letter in the same file are significantly different (*p* < 0.005).

**Table 4:** Nutritional components in cookies with wheat and *P. serotine* defatted flour.

Component (%)	Fc	F3	F4
Fat	68.08 ± 0.29 ^a^	61.94 ± 0.27 ^a^	58.48 ± 0.27 ^a^
Protein	0.71 ± 0.06 ^c^	12.50 ± 0.03 ^a^	11.79 ± 0.02 ^b^
Crude Fiber	0.00 ± 0.00 ^a^	0.93 ± 0.04 ^a^	0.88 ± 0.03 ^a^
Carbohydrates	31.20 ± 0.05 ^a^	24.63 ± 0.05 ^c^	28.85 ± 0.08 ^b^

The values are the average of three assays ± standard deviations of cookies with wheat and *P. serotine* defatted flour. Mean values labeled with a different letter in the same file are significantly different (*p* < 0.005).

**Table 5:** Soluble protein content in *P. serotine* defatted flour.

Soluble Protein	Protein Content (mg/mL)	Fraction Content with Respect of Total Soluble Protein (%)
Albumin	126.09 ± 3.74 ^a^	76.95 ± 2.29 ^a^
Globulin	22.29 ± 4.20 ^b^	13.60 ± 2.56 ^b^
Prolamin	5.39 ± 0.60 ^d^	3.29 ± 0.37 ^d^
Glutelin	10.09 ± 1.61 ^c^	6.16 ± 0.99 ^c^

The values are the average of three assays ± standard deviations of the soluble proteins in *P. serotine* defatted flour. Mean values labeled with a different letter in the same column are significantly different (*p* < 0.005).

**Table 6:** Treatments to obtain protein content in *P. serotine* defatted flour.

Protein Content in Final Precipitate of *P. serotine* Defatted Flour with NaCl and pH Adjustments.					
Sample	No. of Extractions	Time of Extraction (min)	Flour:NaCl Ratio (pH 11)	pH for Protein Precipitation	Protein Content (%)
*P. serotine* defatted flour	1	30	1:12	4.5	73.47 ± 0.92 ^a, b^
	1	30	1:12	3.0	75.37 ± 2.86 ^a^
	1	30	1:6	3.7	66.30 ± 7.80 ^b^
	1	30	1:6	3.0	70.78 ± 4.50 ^a, b^
	1	30	1:3	3.0	69.15 ± 3.07 ^a, b^
**Protein Content in Final Precipitate of *P. serotine* Defatted Flour with Direct Acidification.**					
**Sample**	**No. of Extractions**	**Time of Extraction (min)**	**pH for Protein Precipitation**		**Protein Content (%)**
*P. serotine* defatted flour	1	30	4.5		63.99 ± 6.63 ^b^
	1	30	3.7		76.66 ± 7.36 ^a, b^
	1	30	3.0		81.99 ± 6.96 ^a^

The values are the average of three assays ± standard deviations of protein extractions from *P. serotine* defatted flour. Mean values labeled with a different letter in the same file are significantly different (*p* < 0.005).

**Table 7:** Interfacial protein concentration in *Ps*PC and whey protein concentrate (WPC) emulsions.

Sample	Mass Fraction (ϕ)	Interfacial Protein Concentration (mg/m^2^)
*Ps*PC	0.2	0.009 ± 0.0
	0.4	0.023 ± 0.0
WPC	0.2	0.015 ± 0.0
	0.4	0.014 ± 0.0

The values are the average of three assay ± standard deviations of interfacial protein concentration in *Ps*PC and WPC emulsions.
